# Tumour-infiltrating cytotoxic T lymphocytes in somatotroph pituitary neuroendocrine tumours

**DOI:** 10.1007/s12020-019-02145-y

**Published:** 2019-12-24

**Authors:** Donato Iacovazzo, Sabrina Chiloiro, Eivind Carlsen, Antonio Bianchi, Antonella Giampietro, Tommaso Tartaglione, Chiara Bima, Maria Elena Bracaccia, Francesca Lugli, Liverana Lauretti, Carmelo Anile, Marco Gessi, Cesare Colosimo, Guido Rindi, Alfredo Pontecorvi, Márta Korbonits, Laura De Marinis

**Affiliations:** 1 Centre for Endocrinology, William Harvey Research Institute, Queen Mary University of London, London, EC1M 6BQ UK; 2 Divisione di Endocrinologia, Fondazione Policlinico Universitario A. Gemelli IRCCS, Università Cattolica del Sacro Cuore, Rome, Italy; 3 Pathology, STHF, Skien, Norway; 4 U.O.C. di Radiologia e Diagnostica per Immagini, Istituto Dermopatico dell’Immacolata, IDI-IRCCS, Rome, Italy; 5 Institute of Neurosurgery, Fondazione Policlinico Universitario A. Gemelli IRCCS, Università Cattolica del Sacro Cuore, Rome, Italy; 6 Institute of Pathology, Fondazione Policlinico Universitario A. Gemelli IRCCS, Università Cattolica del Sacro Cuore, Rome, Italy; 7 U.O.C. Radiologia e Neuroradiologia, Dipartimento di Diagnostica per Immagini, Radioterapia Oncologica ed Ematologia, Fondazione Policlinico Universitario A. Gemelli IRCCS, Università Cattolica del Sacro Cuore, Rome, Italy

**Keywords:** Acromegaly, Pituitary neuroendocrine tumour, Lymphocytes, Macrophages, Endocan

## Abstract

**Introduction:**

Somatotroph pituitary tumours are often resistant to first-generation somatostatin analogues and can invade the surrounding structures, limiting the chances of curative surgery. Recent studies suggested that the immune microenvironment and pro-angiogenic factors can influence neuroendocrine tumour prognosis. In this study, we aimed to investigate the prognostic role of immune cell-specific markers and endocan, a proteoglycan involved in neoangiogenesis and cell adhesion, in a cohort of acromegaly patients who underwent pituitary surgery as first-line treatment.

**Subjects and methods:**

Sixty four eligible subjects were identified. CD4+, CD8+ and CD68+ cells and endocan expression were evaluated by immunohistochemistry and results correlated with clinical and neuroradiological findings. Responsiveness to somatostatin analogues was assessed in patients with persistent disease following surgery.

**Results:**

The number of CD8+ lymphocytes was significantly lower in tumours with cavernous sinus invasion (median 0.2/HPF, IQR: 2.2) compared with those without cavernous sinus invasion (median 2.4/HPF, IQR: 2.3; *P* = 0.04). Tumours resistant to first-generation somatostatin analogues had lower CD8+ lymphocytes (median 1/HPF, IQR: 2.4) compared with responders (median 2.4/HPF, IQR: 2.9; *P* = 0.005). CD4+ lymphocytes were observed sporadically. The number of CD68+ macrophages and the endothelial or tumour cell endocan expression did not differ based on tumour size, cavernous sinus invasion or treatment responsiveness.

**Conclusions:**

Our study suggests that a lower number of CD8+ lymphocytes is associated with cavernous sinus invasion and resistance to treatment with first-generation somatostatin analogues in acromegaly patients. These results highlight a potential role of the tumour immune microenvironment in determining the prognosis of somatotroph pituitary tumours.

## Introduction

Somatotroph pituitary neuroendocrine tumours (PitNETs) [1] are, in most cases, benign, although they can be locally invasive, limiting the chances of radical surgical resection, and are often resistant to medical treatment with first-generation somatostatin analogues (SSAs) [2–4]. A significant proportion of these tumours behave aggressively, with rapid growth and high recurrence rates, and the management of these patients is often challenging. For patients with somatotroph PitNETs, various prognostic factors have been suggested, including a younger age and higher GH values at diagnosis, tumour hyperintensity signal in T2-weighted magnetic resonance imaging, somatostatin receptor expression, higher Ki-67 expression and the tumour cytokeratin pattern [5–8]. Recent studies suggested that neoangiogenesis and the tumour immune microenvironment play a role in determining the prognosis of neuroendocrine tumours [9–15]. Tumour-associated macrophages (TAMs), dendritic cells and tumour infiltrating lymphocytes (TILs) are frequently detected in solid tumours, and are involved in the recognition of tumour antigens, in the inflammatory response to tumour cells and in chemotaxis [16]. However, the phenotype and functional activities of both TILs and TAMs are complex. Some TILs behave as effector cells, such as CD8+ and natural killer lymphocytes [17], inducing a cytotoxic cascade resulting in tumour cell death, while other TILs show a regulatory role inhibiting the anti-tumour activity of effector T cells [18]. Similarly, macrophages can be differentiated into M1 and M2 macrophages, with TAMs generally thought to resemble M2-polarised macrophages, which play a role in promoting tumour cell proliferation and progression as well as inhibiting immune response mediated by T lymphocytes [19]. Despite extensive studies on the tumour immune microenvironment in many solid malignancies, little data are available on PitNETs and other neuroendocrine tumours.

Pro-angiogenic factors, such as endocan (also known as ESM1, endothelial cell-specific molecule 1) and the vascular endothelial growth factor (VEGF), seem to play a role in determining PitNET prognosis [9–12]. Endocan is an endothelium-derived proteoglycan involved in cell adhesion and neoangiogenesis. Although endocan is also expressed in normal tissues [20], the expression of endocan and its circulating levels are positively associated with a worse prognosis and poor survival in various cancers [21–23]. Interestingly, endocan has been shown to enhance the adhesion between monocytes and endothelial cells [24], and *Esm1* knock-out mice show decreased vascular permeability and leucocyte extravasation [25], suggesting that endocan expression in endothelial tumour cells could potentially facilitate the trafficking of immune cells to the tumour microenvironment.

In this study, we aimed to assess the role of immune cell infiltration and of endocan expression in a cohort of patients with somatotroph PitNETs who underwent surgery as first-line treatment. We evaluated the expression of immune cell-specific markers (CD4, CD8 and CD68) and endocan by immunohistochemistry and correlated these findings with clinical, imaging and histopathological features and we assessed their prognostic role in correlation with tumour invasiveness and responsiveness to SSAs.

## Subjects and methods

### Patients

The study population consisted of 64 patients with available archival formalin-fixed tissue who were identified retrospectively from a series of 87 acromegaly patients operated between 2000 and 2014. Patients who received medical treatment prior to surgery were excluded. Thirty-five of the 64 patients were included in a previous study from our group [8]. Disease activity was assessed 4–8 weeks after surgery, and persistence of disease was defined as the presence of elevated age-adjusted IGF1 levels and/or lack of suppression of GH levels during the oral glucose tolerance test below 0.4 ng/ml [26]. Patients with persistent acromegaly were subsequently treated with long-acting first-generation SSAs (octreotide LAR or lanreotide ATG). Treatment was started at 20 mg/4 weeks for octreotide LAR and 90 mg/4 weeks for lanreotide ATG, and titrated up, if needed, on the basis of the GH and IGF1 levels. Responsiveness (random GH < 1 ng/ml and normal age-matched IGF1) and partial responsiveness (>50% decrease of both GH and IGF1 levels without normalisation) to SSAs were assessed after at least 6 months of continuous treatment on a stable dose of SSAs. For patients whose disease remained uncontrolled on first-generation SSAs, alternative treatment choices were made. The study was approved by the local Ethics Committee and written informed consent was obtained from all patients.

### Laboratory evaluations

GH and IGF1 were measured using chemiluminescent immunometric assays (Immulite 2000, Siemens Healthcare, Erlangen, Germany). The standard for GH was IS 80/505 until 2010 and IS 98/574 afterwards. The standard for IGF1 was IS 02/254. Inter and intra-assay coefficients of variation were below 5% for both assays.

### Imaging studies

The maximum tumour diameter was measured based on preoperative MRI scans. Cavernous sinus invasion was assessed using the Knosp’s classification; grades 3 and 4 defined cavernous sinus invasion [27]. Invasion of the sphenoid sinus was also evaluated.

### Immunohistochemistry studies

CD4, CD8, CD68, endocan, somatostatin receptor type 2 (SSTR2), Ki-67 and the cytokeratin pattern were evaluated by immunohistochemistry. Source and dilution of the primary antibodies are reported in Table 1. The immunohistochemistry for Ki-67, cytokeratin and SSTR2 was performed manually. Briefly, the sections were dewaxed, rehydrated and antigen retrieval was performed by heating the sections in citrate buffer (pH 6) for 12 min in a microwave oven at 650 W. Primary antibodies were incubated at room temperature for 30 min. Following incubation with a species-specific biotinylated secondary antibody (Vector Laboratories, Burlingame, California, USA), the sections were incubated with the avidin/biotin complex (Vector Laboratories) and the reactions visualised using DAB as a chromogen (Vector Laboratories). For CD4, CD8, CD68 and endocan, the automated Ventana system (Ventana Medical Systems, Oro Valley, Arizona, USA) was employed. Appropriate positive control slides (normal tonsil for CD4, CD8 and CD68; a breast cancer sample for endocan and the normal pituitary gland for SSTR2) were included for each staining, while one section was processed with omission of the primary antibody as negative control. Images were obtained using a whole-slide scanner (3DHISTECH Ltd, Budapest, Hungary) and the immunohistochemical expression was scored by two observers (DI and EC) using the CaseViewer software (3DHISTECH Ltd). In case of discordant results (<10% of all stainings), each case was re-discussed until an agreement could be found.

**Table 1:** Details of the primary antibodies used for immunohistochemistry

Antibody (clone)	Product code	Dilution	Supplier
Ki-67 (MIB-1) mouse monoclonal	F7268	1:75	Dako, Glostrup, Denmark
Cytokeratin (CAM5.2) mouse monoclonal	345779	prediluted	Becton Dickinson, Franklin Lakes, NJ, USA
SSTR2 (UMB-1) rabbit monoclonal	ab134152	1:500	Abcam, Cambridge, UK
Endocan mouse monoclonal	ab56914	1:800	Abcam, Cambridge, UK
CD4 (EPR6855) rabbit monoclonal	ab133616	1:100	Abcam, Cambridge, UK
CD8 (C8/144B) mouse monoclonal	M7103	1:100	Dako, Glostrup, Denmark
CD68 (KP1) mouse monoclonal	M0814	1:800	Dako, Glostrup, Denmark

The number of CD4+ and CD8+ lymphocytes and CD68+ macrophages was expressed as the average of five random high-power fields (HPFs), as previously suggested [28]. Intravascular positive cells were not counted. The expression of endocan was assessed both in endothelial and tumour cells. Considering that the endothelial expression of endocan is often focal in PitNETs [9], at least five HPFs were evaluated for each case, and the endothelial expression was scored as positive if at least one positively stained vessel was observed. The tumour cell expression of endocan was scored semi-quantitatively by multiplying the intensity of the staining (0–3) by the percentage of positive cells (0–100%) to create an *H*-score (range 0–300) [29]. For Ki-67, the percentage of positive cells was calculated, in each case, as the average of five representative areas counting at least 1000 cells/area [5]. Tumours were classified as sparsely granulated, densely granulated or with intermediate phenotype based on the cytokeratin pattern, as previously described [30]. SSTR2 expression was scored taking into account both the subcellular localization and the extent of the staining: score 0, no immunoreactivity; score 1, cytoplasmic immunoreactivity; score 2, membranous staining in less than 50% of cells or incomplete membranous staining; and score 3, circumferential membranous staining in more than 50% of tumour cells [8, 31, 32].

### Statistics

Descriptive statistical analyses included median and interquartile ranges (IQR) for continuous variables. The Mann–Whitney and Kruskal–Wallis tests and Spearman correlation were used for continuous variables. Nonparametric tests were employed because the data were not normally distributed. For qualitative variables, absolute and relative frequencies are reported and the Χ^2^ test was applied. Statistical significance was assumed for *P* < 0.05. A multiple logistic regression analysis was performed in order to identify factors associated with responsiveness to SSAs. Age, gender and variables found to have a *P* < 0.05 at univariate analysis were included in the multivariate analysis. The data were analysed using the SPSS Software, version 22.

## Results

### Patients and outcomes

The study population consisted of 43 females (67.2%) and 21 males (32.8%). Median age at diagnosis was 41.5 years (IQR: 17). Fifty-eight patients (90.6%) harboured macroadenomas. Invasion of the cavernous sinus was detected at diagnosis in 13 cases (20.3%) and of the sphenoidal sinus in two (3.1%). Out of the 64 enroled patients, 40 patients were affected by persistent acromegaly after surgery and, consequently, 21 patients were treated with octreotide LAR and 19 patients with lanreotide ATG. Twenty-six out of the 40 patients treated with first-generation SSAs were considered resistant and were treated with a second-line treatment, including the GH receptor antagonist pegvisomant (*n* = 14), pasireotide LAR (*n* = 10), repeat pituitary surgery (*n* = 5) or radiotherapy (*n* = 2). Median follow-up was 142 months (IQR: 95).

### Immune cell infiltration and endocan expression

**Fig. 1:**
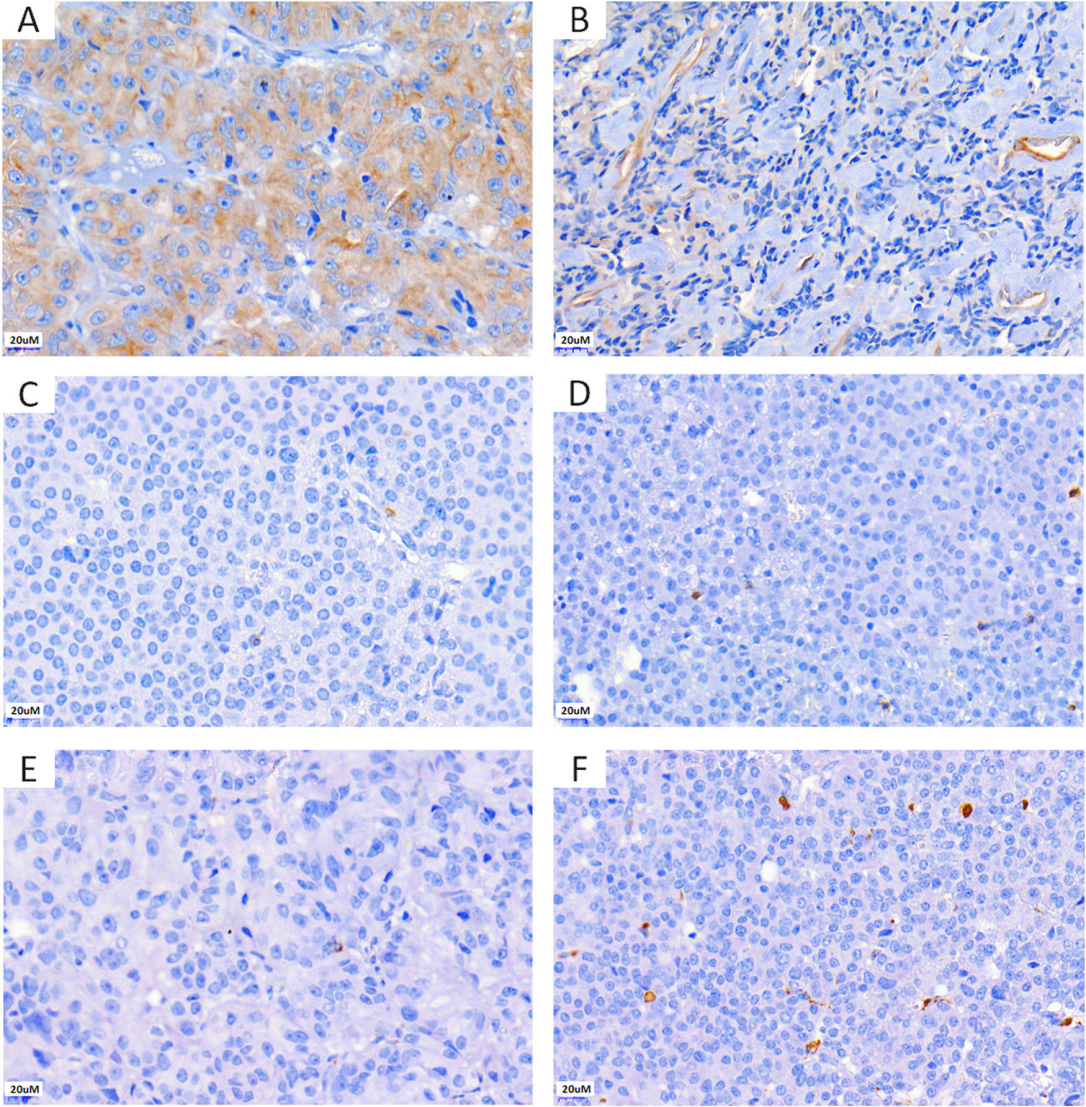
Representative pictures of the immunohistochemistry for endocan and the immune cell markers CD8 and CD68 in somatotroph pituitary neuroendocrine tumours. Immunohistochemistry for endocan showing moderate cytoplasmic expression in the tumour cells only (*H*-score 150) (**a**) and endothelial cell expression with weak tumour cell staining (*H*-score 70) (**b**). Immunohistochemistry for CD8 (**c**, **d**) and CD68 (**e**, **f**): tumours with sparse positive cells (**c**, **e**) and higher immune cell infiltrate are shown (**d**, **f**)

CD8+ cells were observed in 53/64 cases (82.8%), with a median number of 1.4/HPF (IQR: 2.4). CD68+ macrophages were found in 61/64 cases (95.3%) with a median of 5.7 cells/HPF (IQR: 9.3). The number of CD8+ lymphocytes was positively correlated with the number of CD68+ macrophages (*r*: 0.29 *P* = 0.02). As CD4+ lymphocytes were only observed sporadically and in a minority of cases (11/64), this was not investigated further. Endothelial expression of endocan was detected in 26 cases (40.6%), while 38/64 (59.4%) did not show positivity for endocan in the tumour vessels. The median expression of endocan in tumour cells was 110 (IQR: 110). Representative immunostainings are shown in Fig. 1. Tumours with endothelial expression of endocan presented higher number of CD8+ lymphocytes [endothelial endocan positive 1.8 cells/HPF (IQR: 3) vs endothelial endocan negative 1.2 cells/HPF (IQR: 2.4); *P* = 0.03] and CD68+ macrophages [endothelial endocan positive 9.5 cells/HPF (IQR: 9.8) vs endothelial endocan negative 4.3 cells/HPF (IQR: 9.3); *P* = 0.01].

### Correlation with clinical and neuroradiological findings

The number of CD8+ lymphocytes was significantly lower in tumours with invasion of the cavernous sinus (median 0.2 cells/HPF, IQR: 2.2) as compared with those without cavernous sinus invasion (median 2.4 cells/HPF, IQR: 2.3; *P* = 0.04). No significant difference in the number of CD8+ lymphocytes was identified among tumours with invasion of a single or both cavernous sinuses.

The expression of endocan in endothelial cells and pituitary tumour cells and the number of CD8+ lymphocytes and CD68+ macrophages did not differ based on patients’ age, gender, tumour size or GH and IGF1 levels at diagnosis. Similarly, the expression of endocan in endothelial cells and tumour cells and the number of CD68+ macrophages did not differ in PitNETs invading the cavernous sinuous compared with non-invasive ones.

The number of CD8+ lymphocytes was significantly lower in patients resistant to first-generation SSAs (median 1/HPF, IQR: 2.4) as compared with patients responsive to treatment (median 2.4/HPF, IQR: 2.9; *P* = 0.005) (Table 2). No difference was observed in the number of CD8+ lymphocytes among tumours with different SSTR2 expression [score 0–1 median CD8+ lymphocytes 0.2/HPF (IQR: 1.4), score 2 median 1.4/HPF (IQR: 1.6) and score 3 median 1.7/HPF (IQR: 2.4); *P* = 0.26]. Similarly, the number of CD8+ lymphocytes did not differ between sparsely granulated (median 1.2/HPF, IQR: 2) and densely granulated tumours (including tumours with an intermediate cytokeratin pattern) (median 1.8/HPF, IQR: 2.5; *P* = 0.18). The expression of endocan in endothelial cells and pituitary tumour cells and the number of CD68+ macrophages did not differ in tumours responsive to first-generation SSAs as compared with resistant ones (Table 2). A logistic regression analysis confirmed that the only determinants of resistance to first-generation SSAs were a lower age at diagnosis (OR: 1.4, 95%IC: 1–2.1; *P* = 0.04), cavernous sinus invasion (OR: 1.8, 95%IC: 1.3–2.6; *P* = 0.005), higher Ki-67 (OR: 8.3, 95%IC: 1.9–36.4; *P* = 0.03) and lower number of CD8+ lymphocytes (OR: 1.9, 95%IC: 1.2–3.2; *P* = 0.001).

**Table 2:** Clinical, radiological and pathological features of patients responsive and resistant to first-generation somatostatin analogues (SSAs)

	Patients responsive to first-generation SSAs	Patients resistant to first-generation SSAs	*P* value
Gender			
M/F	6/8	5/21	0.1
Median age at diagnosis, years (IQR)	47.5 (28.2)	37 (13.5)	**0.01**
Median GH at diagnosis, ng/ml (IQR)	24.5 (69.7)	23.2 (40.3)	0.3
Median IGF1 × ULN at diagnosis (IQR)	2.6 (1.4)	2.3 (1.3)	0.3
Tumour size			
Macroadenoma/microadenoma	13/1	26/0	0.2
Cavernous sinus invasion			
No	14	14	**0.001**
Single sinus	0	10	
Both sinuses	0	2	
Knosp right cavernous sinus			
Grade 3	0	2	NA
Grade 4	0	2	
Knosp left cavernous sinus			
Grade 3	0	7	NA
Grade 4	0	5	
Median GH after pituitary surgery, ng/ml (IQR)	4.6 (4.6)	4.1 (7.4)	0.6
Median IGF1 × ULN after pituitary surgery (IQR)	1.5 (2.2)	1.4 (0.7)	0.9
Cytokeratin pattern			
Densely granulated or intermediate features	9	11	0.36
Sparsely granulated	5	14	
Negative	0	1	
Median Ki-67, % (IQR)	0.2 (0.5)	0.9 (0.8)	**0.01**
Median CD8 + lymphocytes/HPF (IQR)	2.4 (2.9)	1 (2.4)	**0.005**
Median CD68 + macrophages/HPF (IQR)	7.8 (12)	4.4 (8)	0.9
Median endocan expression in tumour cells, *H*-score (IQR)	100 (112)	100 (90)	0.6
Endocan expression in endothelial cells			
Positive/negative	4/10	9/17	0.4

*ULN* upper limit of normal, *NA* not applicable, *HPF* high-power field

## Discussion

In this study, we investigated the role of the lymphocyte and macrophage infiltrate and the expression of the pro-angiogenic factor endocan in a cohort of somatotroph PitNETs. Our results show that the number of CD8+ lymphocytes is lower in tumours invading the cavernous sinus as well as in tumours resistant to first-generation SSA treatment.

Data on the role of specific immune cell populations in determining the behaviour of pituitary tumours are limited. A study on a cohort of 291 PitNET patients showed that TILs were more abundant in PitNETs compared with the normal pituitary gland and that TILs were associated with a poorer clinical outcome, although a pan-lymphocyte marker was employed, limiting further conclusions [33]. Another study on 35 PitNETs, including 18 somatotroph tumours, showed a positive correlation between the number of infiltrating CD68+ macrophages and tumour size and invasiveness [34]. In the same study, somatotroph tumours were shown to have a higher number of infiltrating CD4+ and CD8+ TILs compared with non-somatotroph tumours. A more recent study investigating CD8+ TILs in 191 patients with PitNETs showed a correlation between CD8+ TILs and circulating GH levels, although potential correlations with tumour invasiveness or response to treatment were not investigated [35]. The anti-tumour activity of CD8+ TILs and their role in mediating the effects of cancer immunotherapy by programmed death 1 (PD-1) blockade are well described [36]. Both PD-1 and its ligand PD-L1 are expressed in PitNETs [35], especially in functioning tumours, and a recently published case report showed a marked response of an ACTH-secreting pituitary carcinoma to checkpoint immunotherapy [37]. Despite the number of CD8+ TILs in PitNETs is lower compared with what is found in several malignant tumours, our study is the first to suggest that CD8+ TILs can play a role in determining PitNET invasiveness and provide evidence to support the use of immunotherapy in selected cases of aggressive and treatment-resistant PitNETs. Moreover, tumours resistant to treatment with SSAs showed reduced CD8+ TILs compared with responsive tumours, while the number of CD8+ TILs did not differ in tumours with different SSTR2 expression or between sparsely and densely granulated tumours. These data suggest that the tumour immune microenvironment could affect responsiveness to SSA treatment independently of SSTR expression and the granulation pattern. Further studies will be needed to elucidate the mechanisms underlying this observation.

In our study, we did not find a correlation between the number of CD68+ TAMs and tumour size, invasion of the cavernous sinus or responsiveness to medical treatment. The role of TAMs in tumourigenesis is complex and not completely clarified. For instance, CD68+ TAMs were identified as predictors of favourable prognosis in gastric, colon and prostate adenocarcinomas, but as a predictor of poor prognosis in neuroblastoma, thyroid cancer and non-functioning pancreatic neuroendocrine tumours [38–44]. TAMs can be differentiated into M1 and M2 macrophages, according to the expression of different cytokines [45]. M1 macrophages act by presenting the antigen and inducing the activation of type 1 helper (Th1) CD4+ T lymphocytes. Instead, M2 macrophages induce the activation of CD4+ regulatory T lymphocytes and inhibit the expression of those cytokines that are required for the activation of CD8+ cytotoxic and Th1 CD4+ lymphocytes [45]. One of the limitations of our study is that we did not evaluate the expression of M1- or M2-specific markers, as this could have potentially highlighted a role of a specific macrophage sub-population in determining tumour behaviour.

In order the evaluate the role of neoangiogenesis, we analysed the expression of endocan in our study population. Endocan is a dermatan sulfate proteoglycan whose expression is induced by VEGF and the fibroblast growth factor 2 [46]. Endocan was shown to act both as a target and modulator of VEGF signalling, as shown by reduced vascular outgrowth and VEGF signaling in the retina of *Esm1* knock-out mice [25]. In recent years, endocan has been investigated as a marker of neoangiogenesis [47], and its overexpression, especially within the endothelial cells of tumour vessels, as well as higher circulating endocan levels, have been associated with poor prognosis in malignant tumours, such as breast cancer, non-small cell lung cancer, gastric cancer and hepatocellular carcinoma [21–23, 48]. According to previous reports, the expression of endocan in PitNETs was found to be associated with invasion of the cavernous sinus [11, 12] and higher risk of recurrence [9]. In our study, however, we did not find any correlation between either endocan endothelial or tumour cell expression and tumour size, invasiveness or response to treatment. Previous studies included mostly non-functioning PitNETs, while our study was focused on a homogenous series of somatotroph tumours, potentially suggesting that the role of endocan as a prognostic marker could be different among PitNET subtypes. Interestingly, the endothelial expression of endocan was found to be associated with a higher number of TILs and TAMs, supporting a role for this molecule in mediating leucocyte extravasation [25].

In conclusion, our findings show a prognostic role for CD8+ TILs in determining somatotroph tumour invasiveness and responsiveness to SSA treatment. The evidence that CD8+ TILs are detected in PitNETs and that a higher CD8+ cell infiltrate is associated with a lower rate of invasiveness and lower rate of treatment resistance provide a rationale for the role of immunotherapy in selected cases of treatment-refractory and aggressive PitNETs.
